# Supplementary material for: Patterns of Intron Gain and Loss in Fungi
Source: PLoS Biol. 2004 Nov 30;2(12):e422. doi: 10.1371/journal.pbio.0020422 (PMC532390; doi:10.1371/journal.pbio.0020422)
Supplement: Table S1 — Also available at http://genes.mit.edu/NielsenEtAl/. (4.3 MB ZIP). [file pbio.0020422.st001.zip › NielsenEtAl/html/1329.html]

AN8010.1.NCU06687.1.MG07289.1.FG06822.1


```
 CLUSTAL W (1.82) Multiple Sequence Alignments - Introns Inserted


Sequence 1: NCU06687.1	706 aa
Sequence 2: FG06822.1	704 aa
Sequence 3: MG07289.1	708 aa
Sequence 4: AN8010.1	711 aa
Alignment Length: 712 aa
Number Identitical Residues: 528 aa
Alignment Score (without introns) 22923


MG07289.1 	MAEGGEREPREVKNHILFEIATEVAHRV1GGIYSVIKSKAPVTTAEYGDRYTLIGPLNHT
NCU06687.1	MAHDNR-EPREVKNHLLFEVATEVAHRV1GGIYSVLKSKAPVTTAEYGDRYTLIGPLNHQ
FG06822.1 	MSSGGE-QTRDIKNHLLFEIATEVAHRV1GGIYSVIKSKAPVTTAEYGDRYTLIGPLNHQ
AN8010.1  	MADDQDPPRRDVRNHMLFEIATEVANRV~GGIYSVLKSKAPVTTAEYGERYTLIGPLNRN
          	*: .     *:::**:***:*****:** ******:************:*********: 

MG07289.1 	S0AAVEVEELEPKDPALTATIQSMKDRGIGILYGRWLIEGAPRVLLIDTKTAYKHLDEWK
NCU06687.1	S0AAVEVEELEPSNPELKATIQAMRDRGIGILYGRWLIEGAPRVLLFDTKTAYGYMNEWK
FG06822.1 	S0AAVEVEEMEPTNPEIAATIQSMRDRGVHIVYGRWLIEGAPRVILIDTKTAYGRMDEWK
AN8010.1  	S~AAVEVEELTPSSPAMKETMQSMKERGIEMVYGRWLIEGAPRVLLIDTGTGYKYLDEWK
          	* *******: *..* :  *:*:*::**: ::************:*:** *.*  ::***

MG07289.1 	TDLWNVASIPSPPGDDETNEAIVFGYLVAWFLGE0YVCHEKKKAVIAHFHEWLAGVGLPL
NCU06687.1	TDLWNVASIPSPDNDEETNEAIVFGYLVAWFLGE0FVCHEKRKAVIAHFHEWLAGVALPL
FG06822.1 	ADLWEAASIPSPPGDDETNEAIVFGYLVAWFLGE0FVCHEKKKAVIAHFHEWLAGVALPL
AN8010.1  	GDLWNTAGIPSPASDHETNEAIVFGYLVAWFLGE0YIAHERRRAVVAHFHEWLAGVALPL
          	 ***:.*.**** .*.****************** ::.**:::**:**********.***

MG07289.1 	CKKRRIDVTTIFTTHATLLGRYLCAGSVDFYNNLQWFDVDAEAGKRGIYHRYCIERAAAH
NCU06687.1	TKKRQIDVTTIFTTHATLLGRYLCAGSVDFYNNLQWFDVDAEAGKRGIYHRYCIERAAAH
FG06822.1 	CKRRRIDVTTIFTTHATLLGRYLCAGSVDFYNNLQWFDVDAEAGKRGIYHRYCIERAAAH
AN8010.1  	TKKRHMDLTTIFTTHATLLGRYLCAGSVDFYNNLQYFDVDAEAGKRGIYHRYCIERAAAH
          	 *:*::*:***************************:************************

MG07289.1 	SCDVFTTVSHITAYESEHLLKRKPDGVLPNGLNVTKFAAMHEFQNLHAQSKEKIHDFVRG
NCU06687.1	SCDVFTTVSHITAYESEHLLKRKPDGVLPNGLNVTKFSAMHEFQNLHQQNKEKIHDFVRG
FG06822.1 	ACDVFTTVSHITAFESEHLLKRKPDGVLPNGLNVTKFSAVHEFQNLHQQAKEKIHDFVRG
AN8010.1  	SADVFTTVSHITAFESEHLLKRKPDGVLPNGLNVKKFSAVHEFQNLHSQSKEKINEFVRG
          	:.***********:********************.**:*:******* * ****::****

MG07289.1 	HFYGHYDFEPENTLYFFTAGRYEFRNKGCDMFIESLARLNHRLKASGSKTTVVAFVIMPA
NCU06687.1	HFYGHYDFEPENTLYFFTAGRYEFRNKGVDMFIESLARLNHRLKTAGSKTTVVAFIIMPA
FG06822.1 	HFYGHYDFDVDNTLYLFTAGRYEFRNKGVDMFIESLARLNHRLKASGSKITVVAFIIMPA
AN8010.1  	HFYGHNDFDFDDTLYVFTSGRYEFRNKGVDMFIEGLARLNHRLKSSGSKTTVVAFIIMPA
          	***** **: ::***.**:********* *****.*********::*** *****:****

MG07289.1 	QTSSLTVEALRGQAVVKSLRDTVDVIEKSIGRRIFERSLKWHDGDPLPDEKELITSQDRV
NCU06687.1	QTTSLTVEALKGQAVIKSLRDTVDVIERGIGRRIFERSVKWHEGDPLPEEKELITSQDRV
FG06822.1 	QTTSLTVEALKGQAVIKSLRDTTHVIEQSIGRRLFERSLKWHEGEPLPDEKELISAQDRV
AN8010.1  	QTSSLTVESLKGQAVVKSLRDTIHMIEQGIGKRMYERCLAWKEGDNMPDEKDLITSQDRV
          	**:*****:*:****:****** .:**:.**:*::**.: *::*: :*:**:**::****

MG07289.1 	LLRRRLFAMKRHGLPPIVTHNMVNDSEDPILNQIRRVQLFNHPTDRVKIVFHPEFLNSAN
NCU06687.1	LLRRRLFAMKRHTLPPIVTHNMLNDHEDPILNQIRRVQLFNHPSDRVKIVFHPEFLSSAN
FG06822.1 	LLRRRLFAMKRHGLPPIVTHNMLNDHEDPVLNQIRRVQLFNHPTDRVKVIFHPEFLNSAN
AN8010.1  	LLRRRLFAMKRHTLPPIVTHNMINDSEDPILNQIRRVQLFNQSSDRVKIVFHPEFLNSSN
          	************ *********:** ***:***********:.:****::******.*:*

MG07289.1 	PVLPLDYDDFVRGTHLGVFASYYEPWGYTPAECTVMGVPSITTNLSGFGCYMEELIENSS
NCU06687.1	PVLPLDYDDFVRGTHLGVFASYYEPWGYTPAECTVMGVPSITTNLSGFGCYMEELIENSS
FG06822.1 	PVLPLDYDDFVRGCHLGVFSSYYEPWGYTPAECTVMGVPSITTNLSGFGCYMEELIENSS
AN8010.1  	PVLPLDYDDFVRGTHLGVFPSYYEPWGYTPAECTVMGVPSITTNLSGFGCYMEELIENSS
          	************* *****.****************************************

MG07289.1 	DYGIYIVDRRTKGVDDSVNQLTNCMLEFCQKSRRQRINQRNRTERLSDLLDWKRMGLEYI
NCU06687.1	DYGIYIVDRRSKGVDDSVNQLTQYMFEFTQKSRRQRINQRNRTERLSDLLDWKRMGMEYV
FG06822.1 	DYGIYIVDRRTKGVDDSVNQLTSFMFDFCGKSRRQRINQRNRTERLSDLLDWKRMGMEYV
AN8010.1  	DYGIYIVDRRMKGVDDSVNQLTDFMYNFTLKSRRQRINQRNRTERLSDLLDWKRMGLEYV
          	********** ***********. * :*  **************************:**:

MG07289.1 	KARQLALRRA~YPNSFDG-DDTADFMNSSDLKISRPFSVPGSPRDRAGMMTPGDFASLQE
NCU06687.1	KARQLALRRA~YPTSFNG-EEEEDFIPGVEQKISRPFSVPGSPRDRTGMMTPGDFASLQE
FG06822.1 	KARQLALRRA~YPNSFVGDEEEEDFIPGVEQKISRPFSVPGSPRDRTGMMTPGDFASLQE
AN8010.1  	KARQLALRRA1YPSSFDNQEDYFDVIGGTEQKISRPLSVPGSPRDRSGMMTPGDFASLQE
          	********** **.** ..::  *.: . : *****:*********:*************

MG07289.1 	GREGLSTEDYVSWKLP2--EEEDPDEYTFPLTLGAKQRPSGPASPLDGVHLNGNGN-
NCU06687.1	SHEGLSTEDYVAWKLP2--EEEDPEEYPFPLTLKQRTGPGSPLDSIQGLQLNGTR--
FG06822.1 	GREGLNTEDYVAWKLP2--EEEDPEEYPFPLTLRKQPAPPSPSETVP---VNGTQ--
AN8010.1  	VKEGLSTEDYVAWRLP2TSEEDDAEDHFYPLTLRTKKS-DRPSSPLDRISINGGQSS
          	 :***.*****:*:** ::**:*.::: :****  :     * ..:    :**  .:
```
